# Dehydropeptide Supramolecular Hydrogels and Nanostructures as Potential Peptidomimetic Biomedical Materials

**DOI:** 10.3390/ijms22052528

**Published:** 2021-03-03

**Authors:** Peter J. Jervis, Carolina Amorim, Teresa Pereira, José A. Martins, Paula M. T. Ferreira

**Affiliations:** Centre of Chemistry, University of Minho, Campus de Gualtar, 4710-057 Braga, Portugal; carolinaamorim753@gmail.com (C.A.); pg37045@alunos.uminho.pt (T.P.); jmartins@quimica.uminho.pt (J.A.M.); pmf@quimica.uminho.pt (P.M.T.F.)

**Keywords:** hydrogel, supramolecular, dehydrodipeptide, drug delivery, wound healing, cancer, smart materials, peptidomimetic

## Abstract

Supramolecular peptide hydrogels are gaining increased attention, owing to their potential in a variety of biomedical applications. Their physical properties are similar to those of the extracellular matrix (ECM), which is key to their applications in the cell culture of specialized cells, tissue engineering, skin regeneration, and wound healing. The structure of these hydrogels usually consists of a di- or tripeptide capped on the *N*-terminus with a hydrophobic aromatic group, such as Fmoc or naphthalene. Although these peptide conjugates can offer advantages over other types of gelators such as cross-linked polymers, they usually possess the limitation of being particularly sensitive to proteolysis by endogenous proteases. One of the strategies reported that can overcome this barrier is to use a peptidomimetic strategy, in which natural amino acids are switched for non-proteinogenic analogues, such as D-amino acids, β-amino acids, or dehydroamino acids. Such peptides usually possess much greater resistance to enzymatic hydrolysis. Peptides containing dehydroamino acids, i.e., dehydropeptides, are particularly interesting, as the presence of the double bond also introduces a conformational restraint to the peptide backbone, resulting in (often predictable) changes to the secondary structure of the peptide. This review focuses on peptide hydrogels and related nanostructures, where α,β-didehydro-α-amino acids have been successfully incorporated into the structure of peptide hydrogelators, and the resulting properties are discussed in terms of their potential biomedical applications. Where appropriate, their properties are compared with those of the corresponding peptide hydrogelator composed of canonical amino acids. In a wider context, we consider the presence of dehydroamino acids in natural compounds and medicinally important compounds as well as their limitations, and we consider some of the synthetic strategies for obtaining dehydropeptides. Finally, we consider the future direction for this research area.

## 1. Introduction

Dehydroamino acid residues are commonly employed in peptidomimetic medicinal chemistry strategies [1]. Furthermore, they feature in their own right in many naturally occurring peptides and several medicinally important molecules [2,3,4,5]. This review summarizes the reports of where dehydroamino acid residues have been successfully employed in the development of supramolecular molecular assemblies, including hydrogels, nanoparticles, nanotubes, nanospheres, and other nanostructures. We begin with a general overview of supramolecular hydrogels and related nanostructures; then, we consider the structure and properties of dehydroamino acids and how these may influence the assembly of supramolecular structures.

### 1.1. Supramolecular Peptide Hydrogels

**Figure 1:**
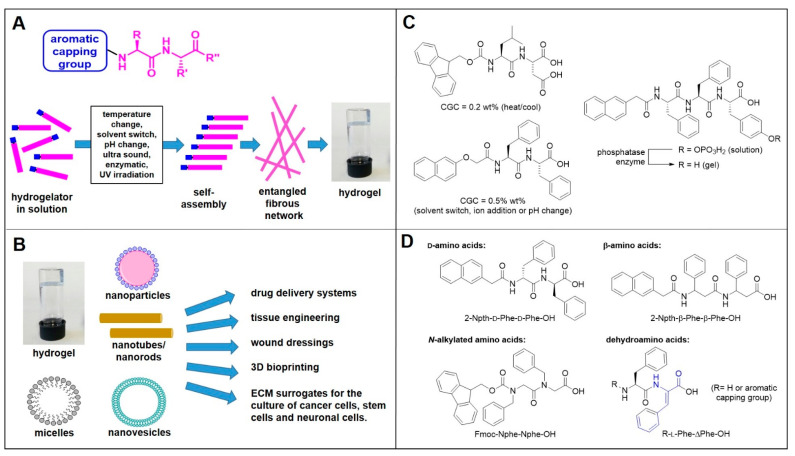
(**A**) Schematic of the formation of peptide hydrogels. (**B**) Biological applications of supramolecular peptide hydrogels and related nanostructures. (**C**) Examples of well-studied supramolecular peptide hydrogelators. (**D**) Some peptidomimetic strategies in the design of supramolecular hydrogels.

Supramolecular hydrogels consist of non-covalently cross-linked polymers and peptides [6]. Peptide-based hydrogels are gaining popularity owing to their intrinsic biocompatibility. Short peptides attached to an aromatic capping group are particularly attractive alternatives as minimalist low-molecular weight gelators [7]. In some specific cases, short peptides without an aromatic capping group can form hydrogels [8,9]. The combination of intermolecular interactions, such as hydrogen bonds, π–π stacking, and van der Waals forces allows molecular self-assembly to take place, with the formation of nanofibrils. When these nanofibrils are able to trap water molecules, self-supporting soft materials, such as hydrogels, may form [6]. The gelation process is often initiated by means of an external trigger, which lowers the solubility of a hydrogelator in solution [10]. The most commonly employed triggers for hydrogelation are the use of a heating–cooling cycle, a pH change, the addition of chelating metal ions, a solvent swap, or an enzymatic, chemical, or photolytic cleavage of a solubilizing group (Figure 1A) [11,12,13,14,15]. A balance of hydrophilic and hydrophobic properties is crucial to the gelation process. If a putative gelator is too hydrophilic, it may stay in aqueous solution, if it is too hydrophobic, then precipitation may occur before the onset of the gelation process [16]. Generally, a clogP value of between 3.4 and 5.5 is considered ideal [7]. In addition to a favorable polarity, two or three aromatic groups are usually required to promote the intramolecular stacking [17]. Hydrogels can be characterized using a variety of techniques, including tube inversion tests, rheology, fluorescence spectroscopy, circular dichroism (CD) spectroscopy, and various microscopic techniques such as scanning electron microscopy (SEM), tunneling electron microscopy (TEM), and atomic force microscopy (AFM) [18].

The properties of hydrogels often resemble those of the extracellular matrix, and as such, they have many biomedical applications in tissue engineering, skin regeneration and wound healing, 3D bioprinting, and biosensors [19]. Alongside other related nanostructures, they are also promising materials for delivery vehicles in sustained and/or targeted drug release (Figure 1B) [20]. The most commonly studied hydrogelators consist of di- or tripeptides attached at the *N*-terminus to an aromatic group such as Fmoc or naphthalene (Figure 1C) [21]. The free carboxylic acid group at the *C*-terminus provides a handle for dissolving a suspension of hydrogelator, as the addition of a base, such as a dilute solution of sodium hydroxide, provides the more aqueous-soluble carboxylate salt. A subsequent reduction of pH using dilute acid protonates the carboxylate back to the neutral carboxylic acid, reducing the solubility and initiating the gelation process. As an alternative to direct mineral acid addition, the group of Adams pioneered the use of glucono-δ-lactone (GdL) as a method for controlled pH reduction, *via* its slow hydrolysis to gluconic acid in the presence of water [22]. More uniform gel structures are obtained in this way, as the rate of pH decrease is now slower than the rate of diffusion. The group of Webber recently demonstrated that the rate of GdL-induced pH change can be used to tune the morphology of supramolecular nanostructures of discotic trimeric peptides [23].

Some important examples of hydrogelators are shown in Figure 1C, but countless more have been described. These are presented within many detailed reviews published in this area [24,25]. Despite the progress being made in this field, there are still areas for improvement. Much of the work in this area has been carried out on fluorenylmethoxycarbonyl(Fmoc)-capped hydrogels, which imparts favorable gelation properties. However, with regard to biological applications, there are concerns over the stability of these hydrogelators owing to the base-lability of the Fmoc group and the toxicity associated with the degradation products of the Fmoc group [26,27]. For example, the group of Thordarson studied the leaching of monomers from a Fmoc-Phe-Phe-OH hydrogel into the surrounding medium, and they found that the degraded hydrogel was cytotoxic to various cell lines [27]. Furthermore, the peptide chain itself, consisting of canonical amino acids, is susceptible to proteolytic degradation by endogeneous enzymes in vivo [28]. This has prompted research groups to search for alternative structures. Peptidomimetic strategies, where the standard canonical amino acids have been replaced by non-coding amino acids, are gaining interest in this area, as these are not recognized by naturally occurring enzymes in biological systems. The most commonly employed strategies involve the use of D-amino acid, β-amino acid, *N*-alkylated amino acid, α-aminobutyric acid, and dehydroamino acid residues [29,30]. The group of Xu investigated hydrogelators containing D-amino acids, and they found increased resistance to protease enzymes [31]. The group of Xu also investigated hydrogelators constructed from β-amino acids, again with favorable results [32]. The group of Nilsson investigated *N*-benzylated glycine residues as replacements for phenylalanine residues (Figure 1D) [33]. This review focuses on hydrogelators and nanostructures containing a dehydroamino acid residue, the presence of which not only increases enzymatic stability but also significantly alters the structural conformation and overall properties of the peptide.

### 1.2. Structure of Dehydroamino Acids and Dehydropeptides

**Figure 2:**
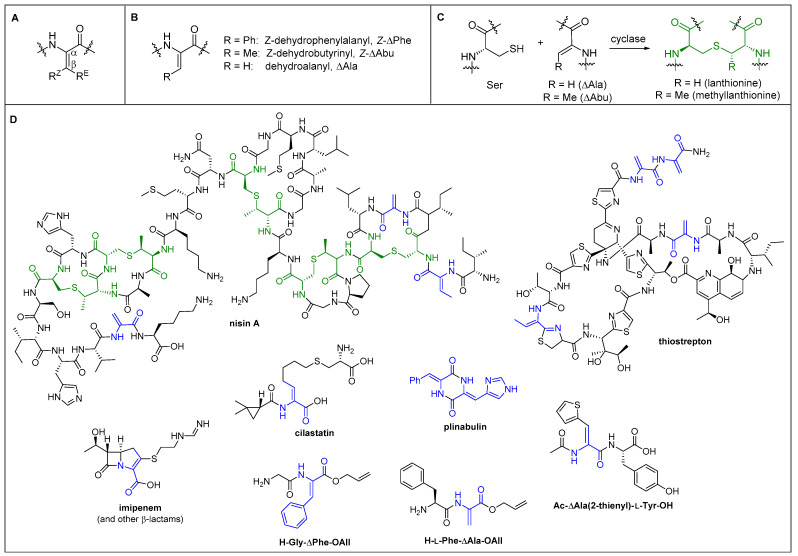
(**A**) General structure of dehydroamino acid residue. (**B**) Important dehydroamino acid residues relevant to supramolecular hydrogels and nanostructures. (**C**) Lanthionine residues are formed through an intramolecular reaction involving serine and dehydroalanine, in the biosynthesis of lantibiotic peptides. (**D**) Examples of biologically active dehydropeptides.

Dehydroamino acid residues differ from their corresponding canonical proteinogenic amino acids by the presence of unsaturation (i.e., a double bond), which is usually between the carbons C-α and C-β. When the double bond is in this position, they are known as α,β-didehydro-α-amino acids. Although alternative positional isomers can be invoked, for the purpose of this review, α,β-didehydro-α-amino acids and α,β-didehydropeptides shall be simply referred to as dehydroamino acids and dehydropeptides, respectively. The presence of the double bond has a number of effects. Structurally, the planar geometry around the double bond means that the stereogenic center of the corresponding canonical amino acid is no longer present. Molecular flexibility is restricted, with fixed bond angles around the C-α and C-β carbon atoms. The overlapping p-orbitals ensure that bond rotation around Cα=Cβ is completely suppressed, and therefore, if R^1^ and R^2^ are different, then two possible geometric isomers are possible, namely *E* and *Z*. The *E* isomer features the substituent *cis* to the carbonyl, whereas in the *Z* isomer, the substituent is *cis* to the nitrogen atom (Figure 2A). In dehydroamino acids, the *Z* form is the more thermodynamically stable on steric grounds [34]. Synthetic methods exist for accessing both possible isomers (*vide infra*), although methods producing the *Z*-isomer are more commonly reported. The most important dehydroamino acid residues involved in supramolecular structures are dehydrophenylalanine, dehydroalanine, and dehydro-2-aminobutyric acid (Figure 2B).

The presence of the double bond within a peptide chain can have a profound effect on the secondary structure, often predictably, compared with the corresponding peptides comprised of solely canonical amino acids. The group of Broda studied the effects of the dehydroamino acid residue double bond geometry on the secondary structure of short peptides by comparing the conformations of Boc-Gly–*E*-ΔPhe-NHMe and Boc-Gly–*Z*-ΔPhe-NHMe. They found that the geometry has a profound effect on the secondary structure, with the *E*-isomer far less likely to adopt β-turn conformations [35]. The effect of dehydroamino acid residues on the structure and stability of helical tetrapeptides has been investigated by Joaquin et al., who found that the position of a dehydroamino acid residue within a tetrapeptide has a substantial effect on the secondary structure [36]. The conformations of dehydropeptides have also been extensively studied by the group of Chauhan [37,38,39]. Perhaps most significantly, they found that switching a parent phenylalanine residue for a *Z*-dehydrophenylalanine (*Z*-ΔPhe) prevents β-sheet formation (i.e., “β-breakers”), suggesting a role in inhibiting the amyloid formation and fibrillization implicated in Alzheimer’s disease [40].

### 1.3. Occurrence in Nature and Pharmaceutics

The purpose of this section is to demonstrate that dehydroamino acid residues can serve as biocompatible and non-cytotoxic structural motifs, which as well as providing increased enzymatic stability also offer their own unique biological activities. We will also address some of their limitations. In addition to conventional dehydroamino acid residues, other relevant motifs include lanthionine and methyllanthionine residues, which are special cases where the thiol group of a serine residue reacts intramolecularly (via a dedicated cyclase enzyme) with ΔAla or ΔAbu, to form a thioether bridge (Figure 2C) [41].

The dehydroamino acid motif is frequently encountered in nature, usually as constituents of large peptides [2,42]. In these cases, the main amino sequences are ribosomally assembled, and then the unsaturation is introduced post-translation. They are most commonly produced by bacteria, followed by fungi. For example, dehydroalanine, dehydro-2-aminobutyric acid, lanthionine, and methyllanthionine residues are constituents of nisin A, which is a polycyclic antibacterial and food preservative produced by *Lactococcus lactis* [43]. Thiostrepton is an oligopeptide antibiotic used in veterinary medicine that is produced by various *Streptomyces* bacteria, which contains ΔAla and ΔAbu [4]. The dehydroamino acid motif is also present in medicinally important small molecules, such as several classes of β-lactam antibiotics, for example cephalosporins, oxacephems, cephamycins, carbapenems, and penems [44]. The carbapenem class includes thienamycin and its more stable analogue, imipenum. Imipenum is hydrolyzed by renal dehydrogenase I enzyme in the liver, and therefore, it is co-administered with another dehydropeptide, cilastatin, which acts as a dehydrogenase inhibitor [5,45]. Plinabulin, a cyclic dipeptide containing a dehydrophenylalanine residue and a dehydrohistidine residue, is currently involved in world-wide phase 3 clinical trials for non-small cell lung cancer (Figure 2D) [3].

There are some examples in the literature of biologically active synthetic dehydrodipeptides. Schorlemmer et al. reported that acetyldehydro-3-*Z*-(2-thienyl)alanyltyrosine (Ac-ΔAla(2-thienyl))-l-Tyr-OH) and acetyldehydro-3-(2-furyl)alanyltyrosine (Ac-ΔAla(2-furyl))-l-Tyr-OH) were able to activate macrophages in mice. Following in vivo activation, the macrophages were able to kill several tumor cell-lines in vitro [46]. Latajka et al., and then later Makowski et al., reported that various short dehydropeptide esters and amides are capable of inhibiting cathepsin B enzymes, which are implicated in several inflammatory diseases (Figure 2D) [47,48,49].

### 1.4. Pharmacological Considerations

α,β-Unsaturated carbonyl groups are potentially electrophilic in reactivity at C-β by means of conjugate addition. Oxy-Michael, aza-Michael, and thia-Michael reactions can occur by reaction with alcohols, amines, and thiols, respectively. In biological settings, the reaction is particularly selective for “soft” nucleophiles, such as thiols, which in nature are provided by the side chain of cysteine. Michael acceptors can be a challenge for medicinal chemists to work with if this reactivity is undesired. Sometimes, they can provide false hits in screening assays due to off-target binding, be deactivated in vivo through their reaction with cellular thiols such as glutathione (GSH), or exhibit increased cytotoxic effects [50]. On the other hand, medicinal chemists are often able to exploit this reactivity if a cysteine (thiol-containing) residue is present within the target enzyme active site in a strategy known as “targeted covalent inhibition”, which most notably is often able to overcome the problem of drug resistance.

In the context of dehydropeptides, we have already seen that ΔAla and ΔAbu can undergo intramolecular thio-Michael reactions in the formation of lantibiotics, by the action of specific cyclases (Figure 2C). Generally, Michael reactivity decreases with the presence of electron-donating β-substituents, such as alkyl groups or electron-rich aromatics. Electron-withdrawing substituents can increase the reactivity, but the rate of the reverse reaction (retro-Michael) is also increased [50]. With this in mind, the group of Joaquin et al. examined the reactivity of dehydroamino acid residues present in various tetrapeptides [36]. They found that peptides containing ΔAla were reactive to nucleophiles, e.g., the thiol group of cystamine. The residues *Z*-ΔAbu and ΔVal were resistant to nucleophilic attack, presumably on steric grounds, suggesting that β-substituted dehydroamino acid residues are more suited to in vivo applications. 

## 2. Dehydropeptide Hydrogels and Other Nanostructures

In the structures of reported dehydropeptide hydrogels and nanostructures, the geometry around the Cα=Cβ double usually exists as the *Z* geometric isomer, which is the most thermodynamically stable isomer and the one for which the most synthetic methods of preparation exist [34]. Often, reports of dehydropeptide hydrogels do not explicitly state the *Z*/*E* stereochemistry present or provide proof of the stereochemistry. In these cases, the structures are shown, inferring the stereochemistry from the known outcome of the synthetic method employed (if possible) whilst omitting the stereo-descriptor from the text of the short-hand name.

### 2.1. Dehydropeptides without Capping Groups (Free Amine and Acid Groups at N-Terminus and C-Terminus)

#### 2.1.1. Nanostructures from Uncapped Dehydropeptides 

A short time before the first dehydropeptide hydrogelators were reported, the group of Chauhan reported two important discoveries. In 2007, the group reported on the ability of H-l-Phe-ΔPhe-OH to form ordered nanotubes [51]. The structures were longer and thinner than those obtained from the corresponding dipeptide composed of canonical amino acids (H-l-Phe-l-Phe-OH). They were stable to high temperatures, had a large pH range, and non-specific proteases (proteinase K). Around the same time, the same group reported two more dehydrodipeptides, H-l-Lys-ΔPhe-OH and H-l-Glu-ΔPhe-OH, as nanovesicles capable of encapsulating various model drug compounds, including vitamin B12, hemin, insulin, riboflavin, Pf MSP-I19, Pf MSP-3N, Pf HRP-II, BSA, lysozyme, and anti-mouse immunoglobin G (IgG) [52]. These nanovesicles were again stable to various proteases and proved non-toxic to Vero and HeLa cells.

Later studies showed that nanoparticles of H-l-Met-ΔPhe-OH were effective delivery vehicles for curcumin that were superior to H-l-Leu-ΔPhe-OH and H-l-Ile-ΔPhe-OH [53]. Curcumin possesses anti-cancer and anti-inflammatory properties, but as in many hydrophobic drugs, poor aqueous solubility is responsible for low bioavailability. Encapsulation within amphiphilic nanostructures offers a potential solution, and the authors found that when curcumin-loaded nanoparticles of H-l-Met-ΔPhe-OH were dispersed in aqueous solution, bioavailability was improved. This provided an increased cytotoxicity to the cancer cells lines HeLa, MCF-7, and HUH-7, whilst being non-cytotoxic to human fibroblasts (L-929) in concentration up to 50 μM. In animal studies, the loaded nanoparticles delayed tumor growth and enhanced survival rates in B6F10 melanoma mice.

Panda et al. reported that H-l-Arg-ΔPhe-OH and H-l-Lys-ΔPhe-OH were able to bind plasmid DNA and be taken up by cells. The plasmid DNA was resistant to the action of DNase enzymes [54]. A similar result was observed by Khatri et al., who from a panel of dehydrodipeptides found that H-l-Arg-ΔPhe-OH was the best suited for forming spherical nanoparticles capable of delivering plasmid DNA to HEK293T cells [55]. The release was pH dependent, with the plasmid DNA retaining their native conformation at endosomal pH.

Basker et al. functionalized iron oxide nanoparticles with H-l-Arg-ΔPhe-OH for targeting cancer cells in the presence of a pulsed magnetic field, providing heat-induced cell death by altering the membrane permeability of lung cancer cells [56]. This strategy provides a targeted delivery at low cost whilst avoiding problems associated with toxicity and drug resistance.

Varshney et al. attached lactabionic acid (LA) to a dehydrodipeptide to form a H-l-Arg-ΔPhe-LA conjugate [57]. In turn, this was used to create LA-l-Arg-ΔPhe-OH/miR-199a-3p nanoparticles, for delivering microRNA to hepatocellular carcinoma. The conjugated LA is a ligand for asialoglycoprotein receptors, which are over expressed on hepatocytes.

Folic acid receptors are known to be overexpressed on cancer cells, which lead Panda et al. to derivatize nanoparticles of H-l-Arg-ΔPhe-OH with folic acid (FA) in order to provide a tumor-targeted drug delivery of the cancer drug, doxorubicin. Doxorubicin entrapped within FA-l-Arg-ΔPhe-OH nanoparticles was able to provide an enhanced target specificity and anti-tumor effect compared with free doxorubicin or non-folic acid conjugated nanoparticles. The stability and low toxicity of these peptide nanoparticles provides advantages over the alternative types of nanoparticles previously studied, such as polymeric or metallic nanoparticles [58].

The group of Chauhan combined *Z*-dehydrophenylalanine with β-phenylalanine (another non-coding amino acid). The dipeptide H-β-Phe-ΔPhe-OH formed nanotubes with different properties to the related H-β-Phe-l-Phe-OH and H-l-Phe-l-Phe-OH dipeptides. Nanotubes of H-β-Phe-ΔPhe-OH were stable over a range of temperatures, stable to non-specific proteases, and demonstrated non-cytotoxicity to HeLa and L929 cells up to concentrations of 250 μM. The authors found that H-β-Phe-ΔPhe-OH-encapsulated mitoxanthrone was more efficient in killing HeLa and B6F10 cancer cells than free mitoxanthrone [59].

**Figure 3:**
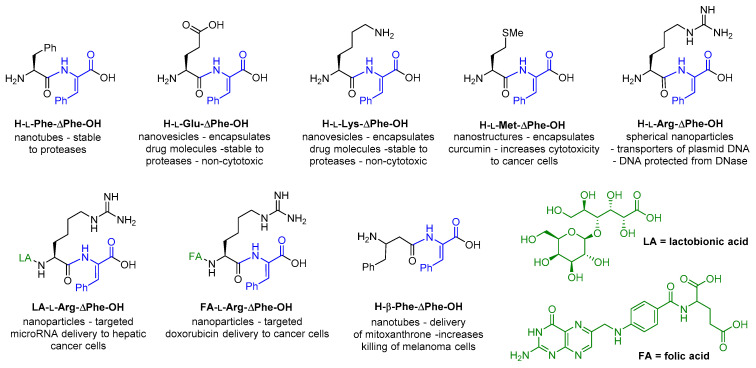
Summary of uncapped dehydrodipeptides capable of forming nanostructures.

The uncapped dehydrodipeptides able to form nanostructures are summarized in Figure 3.

#### 2.1.2. Hydrogels from Uncapped Dehydropeptides 

In 2008, Chauhan reported the first dehydrodipeptide hydrogel [60]. Dehydrodipeptide, H-l-Phe-ΔPhe-OH, was able to form a stable supramolecular hydrogel by adding a 50 mg/mL 1,1,1,3,3,3-hexafluoroisopropanol (HFIP) solution of hydrogelator to 0.8 M sodium acetate buffer, to achieve a final concentration as low as 0.2 wt % at neutral pH. Notably, unlike other known small dipeptide hydrogelators, this compound had no aromatic capping group attached to the *N*-terminus and therefore features a free amine group and carboxylic acid group at the *N*-terminus and *C*-terminus, respectively. With a molecular weight of 310 g/mol, it was one of the smallest known hydrogelators. The conformational constraint conferred by the double bond proved to be crucial to gelation, as the corresponding saturated dipeptide, H-l-Phe-l-Phe-OH, failed to provide a gel under the same conditions. In addition, the double bond imparted proteolytic stability, with the hydrogelator being inert to the action of chemotrypsin and other enzymes present in cell culture supernatant. The hydrogel possessed high mechanical strength whilst being non-toxic to HeLa and L929 mammalian cells in cell viability assays. The hydrogel was able to encapsulate and release model drug compounds, and therefore, it possessed potential for sustained drug delivery. The gel strength increased with increasing salt concentration, and it decreased with increasing temperature above a threshold temperature of 50 °C. The gels were stable at neutral or basic pH, but reducing the pH to below 7 °C caused gel disassembly. Therefore, the hydrogels are responsive to a range of physical conditions (temperature, pH, and salt concentration) and provide an opportunity for tunable drug delivery [60]. 

In 2010, the same team extended the peptide sequence to form the heptadipeptide sequence H-l-Phe-Δ-Phe-l-Arg-Gly-l-Asp-Gly-Gly-OH, which combined the gelation capacity of H-l-Phe-ΔPhe-OH with the cell-adhesion properties of “RGD” (L-Arg-Gly-l-Asp) [61]. The resulting gel was able to support 3D growth and proliferation of mammalian cells (HeLa and L929) for two weeks. The low-toxicity combined with cell growth-promoting properties provides a promising candidate tissue engineering and cell biology applications.

In 2016, the group of Chauhan extended their search for hydrogels based on the H-l-Phe-ΔPhe-OH structure by synthesizing a panel of 16 variants of H-l-AA-ΔPhe-OH (where AA = a canonical amino acid residue) and testing for gelation ability at 0.4 wt % [62]. They found that only H-l-Leu-ΔPhe-OH provided a strong hydrogel. The hydrogel was injectable (i.e., converts between gel and liquid state in response to applied strain), stable to trypsin, non-toxic to HEK293T cells, and was able to encapsulate a range of hydrophilic and hydrophobic drug molecules. The release rate was found to increase with increasing hydrophilicity of the drug molecule. An in vivo study showed that the sustained release of the cancer drug, mitoxanthrone, from the hydrogel was able to reduce tumor growth.

In 2020, Kumar Thota et al. reported hybrid gels of H-l-Leu-ΔPhe-OH and Fo-l-Met-l-Leu-l-Phe (fMLF, a macrophage attractant) as possible wound-healing materials [63]. Co-assembly afforded injectable gels, which were non-toxic to a variety of human cells including HEK293T cells, macrophages, and fibroblasts in viability assays. The gels were also able to act as 3D platforms for the culture of macrophages and fibroblasts. The presence of Fo-l-Met-l-Leu-l-Phe did not alter the secondary structure of gels of the parent gel, and Fo-l-Met-l-Leu-l-Phe retained its ability to attract macrophages, which is beneficial for the wound-healing process. In drug delivery studies, the hydrogel provided a slow release of various antibiotic molecules, including ciprofloxacin, suggesting an ability to prevent infections at the wound site over a prolonged time period.

**Figure 4:**
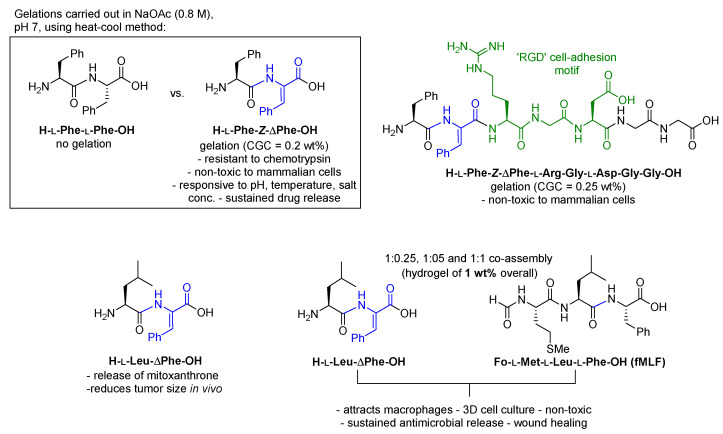
Summary of uncapped short dehydropeptides capable of forming hydrogels.

The uncapped dehydrodipeptides able to form hydrogels are summarized in Figure 4.

### 2.2. Dehydropeptides Modified at the C-Terminus

**Figure 5:**
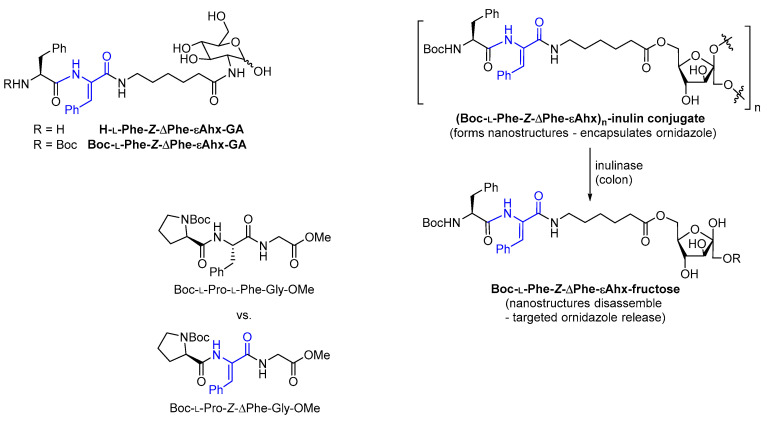
Summary of dehydrodipeptides modified at the *C*-terminus.

In 2012, the group of Kumar Sharma reported glucosamine-conjugated dehydropeptide hydrogelators, H-l-Phe-ΔPhe-εAhx-GA and Boc-l-Phe-ΔPhe-εAhx-GA, as potential drug delivery vehicles (Figure 5) [64]. Conjugation to glucosamine increased the aqueous solubility. At low concentrations (0.1 wt %), the conjugates provide spherical nanostructures of approximately 55 and 175 nm for H-l-Phe-ΔPhe-εAhx-GA and Boc-l-Phe-ΔPhe-εAhx-GA, respectively, determining by techniques such as dynamic light scattering (DLS), AFM, and TEM. At higher concentrations (1.0–2.0 wt %), the conjugates formed hydrogels. Furthermore, the conjugates possessed intrinsic antimicrobial activity, as evidenced by disk diffusion assays. In particular, the conjugates were active against *Micrococcus flavus*, *Bacillus subtilis,* and *Pseudomonas aeruginosa*. Successful drug encapsulation by nanostructures of the peptides (0.5 wt %) was achieved with model hydrophobic dye compounds, namely eosin (0.1 wt %) and *N*-fluoresceinyl-2-aminoethanol (FAE) (0.1 wt %). The peptide containing a free amine group was able to reduce auric chloride to form gold nanoparticles conjugated to the peptide. No cytotoxicity to HeLa cells was observed at concentrations of up to 25 μM.

Then, in 2017, the same team conjugated dehydropeptides to the natural polysaccharide, inulin [65]. By using carbodiimide (CDI) coupling chemistry, the team polyesterified the primary alcohols along the inulin chain to form a dehydropeptide array. The resulting conjugate, (Boc-l-Phe-ΔPhe-εAhx)_n_-inulin, was able to self-assemble into nanostructures of 146–486 nm dimensions, which were able to encapsulate the antibiotic, ornidazole, and then release the drug in a controlled manner. These structures have great potential for targeted drug delivery to treat colonic diseases, as the nanostructures can be disassembled by enzymatic degradation using inulinase, which is produced by colonic bacteria. This degradation of the nanostructures is accompanied by the accelerated release of the drug cargo (Figure 5). Changes to the pH had little effect on the rate of release. In cell viability assays, the nanostructures were non-toxic to HEK293 cells at concentrations of up to 60 μg/mL.

Rekha Deka et al. discovered that tripeptides Boc-l-Pro-l-Phe-Gly-OMe and Boc-l-Pro-*Z*-ΔPhe-Gly-OMe were able to form nanostructures in aqueous solution (Figure 5) [66]. The presence of the dehydrophenylalanine residue was found to have a significant effect on the drug loading ability, as Boc-l-Pro-*Z*-ΔPhe-Gly-OMe was able to encapsulate the antibiotic compound ornidazole and the anti-cancer compound curcumin at higher loading levels than Boc-l-Pro-l-Phe-Gly-OMe. The nanostructures of the dehydropeptide were able to provide a sustained drug release of ornidazole and curcumin of 34% and 23%, respectively, after 6 days. These nanostructures could be further stabilized with D-α-tocopheryl polyethylene glycol (TPGS or vitamin E TPGS), which reduced the overall release of ornidazole and curcumin to 30% and 19%, respectively, over the same time period. In MTT viability assays, the dehydropeptide showed little toxicity to MCF-7 cells up to a concentration of 48 μg/mL.

### 2.3. Dehydropeptides Modified at the N-Terminus

#### 2.3.1. *N*-Conjugated with Non-Steroidal Anti-Inflammatory Drugs (NSAIDs)

Non-steroidal anti-inflammatory drugs function by inhibiting cyclooxygenase (COX) enzymes, which are responsible for the biosynthesis of prostaglandins and thromboxanes responsible for pain and inflammation in response to injury [67]. The unwanted side effects of many NSAIDs means that their administration as prodrugs or as nanoformulations are attractive strategies. COX-mediated inflammation plays a role in tumor development. COX enzymes are also over-expressed on the surface of tumor cells and therefore hold potential for targeted cancer therapy [68]. Important examples of NSAIDs include aspirin, ibuprofen naproxen, and ketoprofen [36]. First, we will briefly mention some of the other examples of peptidomimetic supramolecular hydrogels conjugated to NSAIDs [69]. The group of Xu reported that conjugates of naproxen with peptides of D-amino acids were able to form hydrogels. They were not only stable to proteases (relative to the equivalent conjugates containing L-amino acids) but retained the anti-inflammatory activity of the parent NSAID. More significantly, they provided increased COX-2 vs. COX-1 selectivity, potentially minimizing the gastric side effects of COX inhibitors [70]. The group of Cui reported similar observations for ketoprofen-capped D-amino acid-containing peptides [71]. Majumder et al. investigated conjugates of naproxen with β-amino acids, which also provided increased enzymatic stability whilst retaining the anti-inflammatory properties of naproxen [72]. A naproxen-dipeptide conjugate was very recently identified as a folate receptor ligand from a DNA-encoded chemical library [73].

Our research group synthesized the first dehydropeptides to be conjugated with NSAIDs. The focused library of Npx-l-Phe-ΔPhe-OH, Npx-l-Phe-ΔAbu-OH, Npx-l-Val-ΔPhe-OH, and Npx-l-Ala-ΔPhe-OH were able to form hydrogels, with critical gelation concentrations (CGCs) of 0.4–0.8 wt %. The properties of Npx-l-Phe-ΔPhe-OH were compared with the parent Npx-l-Phe-l-Phe-OH, and it was found that Npx-l-Phe-ΔPhe-OH offers increased resistance to chemotrypsin, a lower CGC (0.4 compared with 0.8 wt %), and a higher pH tolerance [74].

This work was followed up with a more in-depth study focused on Npx-l-Trp-*Z*-ΔPhe-OH and Npx-l-Trp-Z-ΔAla-OH as protease-resistant gelators. Hydrogels of Npx-l-Trp-*Z*-ΔPhe-OH possessed higher elasticity and lower CGCs, whilst Npx-l-Trp-Z-ΔAla-OH possessed self-healing and injectable properties. Both hydrogels showed promise as delivery systems for hydrophobic cancer drugs [75].

In a further study, the peptide structure of a minimum gelator module, Npx-l-Ala-ΔPhe-OH, was extended so as to incorporate L-Arg-Gly-Asp (“RGD”), a cell-binding motif that is overexpressed on cancer cells. Assembled using a combination of solution phase and solid phase peptide synthesis, the resulting construct, Npx-l-Ala-Z-ΔPhe-Gly-l-Arg-Gly-l-Asp-Gly-OH, was an effective nanocarrier [76].

The gelation of the dehydrodipeptides Npx-l-Tyr-Z-ΔPhe-OH and Npx-l-Asp(OH)-Z-ΔPhe-OMe was performed in the presence of superparamagnetic iron oxide nanoparticles (SPIONs) to incorporate the SPIONs within the hydrogel network. The magnetic behavior of the SPIONs was retained within the gel network. These hydrogel composites are stimuli-responsive, as magnetic excitation with AMF generates heat accompanied by a gel-to-sol transition, which may allow targeted drug delivery [77].

The hydrogel of Npx-l-Met-Z-ΔPhe-OH containing core/shell manganese ferrite/gold nanoparticles or gold-decorated manganese ferrite nanoparticles were created, exploiting the affinity of sulfur for gold, and these hydrogel composites showed promising results toward targeted cancer therapy. The dehydroamino acid provided proteolytic stability, while the naproxen capping group is a ligand for COX enzymes, which are overexpressed on cancer cells. The release of the anti-cancer drug, curcumin, was investigated, which was released at a faster rate from irradiated gels compared with non-irradiated gels, and therefore potentially had a dual action in cancer therapy [78].

The increased proteolytic resistance of these naproxen-dehydropeptides, compared with naproxen-dipeptides consisting of canonical amino acids, could potentially affect their ability to release naproxen and act as anti-inflammatory prodrugs. Moreira et al. considered that these conjugates may themselves retain the anti-inflammatory properties of the conjugated NSAID and therefore be suitable as hydrogels for “self-delivery” [79]. With this in mind, the biological activities of a panel of hydrogelator naproxen conjugates was assessed. Many of the examples were found to retain anti-inflammatory activity. The most interesting compound was Npx-l-Ala-*Z*-ΔPhe-OH, which could inhibit enzymes involved in the inflammation process, COX-2 and lipoxygenase (LOX), to a similar extent as the parent naproxen, indicating a dual action. Interestingly, COX-1 inhibition was significantly reduced, meaning that this conjugate is, in fact, a COX-2-selective inhibitor, which is a desired profile for minimizing the side effects of NSAIDs. The compound library was also assessed for alternative biological activities, and Npx-l-Tyr-ΔPhe-OH was found to be an effective proteasome inhibitor. The inhibition of proteasome enzymes is reported to have potential in cancer therapy. A conjugate that can both bind COX enzymes and inhibit proteasome enzymes may be a powerful combination for targeting cancer cells [79].

**Figure 6:**
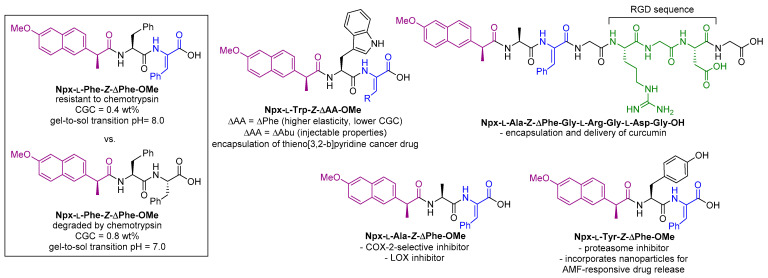
Summary of naproxen-capped peptides capable of forming hydrogels.

Naproxen-capped dehydropeptide hydrogelators are summarized in Figure 6.

#### 2.3.2. *N*-Conjugated with Carboxybenzyl (Benzyloxycarbonyl) Groups

**Figure 7:**
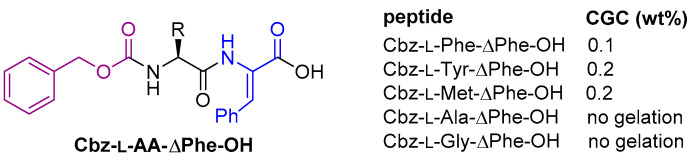
Summary of carboxybenzyl-capped peptides capable of forming hydrogels.

Veloso et al. reported the synthesis of a panel of carboxybenzyl-protected dehydrodipeptides and investigated their hydrogelation properties [80]. Cbz-l-Phe-ΔPhe-OH, Cbz-l-Tyr-ΔPhe-OH, and Cbz-l-Met-ΔPhe were all able to form hydrogels, at 0.1, 0.2, and 0.2 wt %, respectively (Figure 7). Cbz-l-Ala-ΔPhe-OH and Cbz-l-Gly-ΔPhe-OH both failed to provide hydrogels. The successful hydrogelators showed promising drug delivery properties using membrane models. The anti-cancer compounds curcumin and doxorubicin could be encapsulated and then sustainably released. Furthermore, the hydrogelators were found to be non-toxic to the keratynocyte cell line, HaCat, at concentrations of up to 100 μM.

## 3. Synthesis of Dehydropeptides

The synthesis of short peptides containing canonical amino acids is usually a routine procedure for organic chemists, whether achieved by solution-phase or solid-phase synthesis. However, the presence of a dehydroamino acid residue complicates the synthetic process because the nucleophilicity of the amine is reduced by the conjugation of its lone pair of electrons with the double bond. Indeed, this enamine moiety is in equilibrium with the corresponding imine tautomer, which is subject to hydrolysis to the corresponding α-ketocarbonyl. A number of strategies exist for introducing the unsaturation, which have been reviewed by Humphrey [81] and later Bonauer [82]. We shall only briefly consider synthetic methods, focusing on those that are commonly used in the synthesis of dehydrodipeptide hydrogelators or those that appear especially useful for the synthesis of such dehydrodipeptides. The examples discussed here are not intended to be exhaustive but rather serve as an overview of the various reaction classes available.

**Scheme 1:**
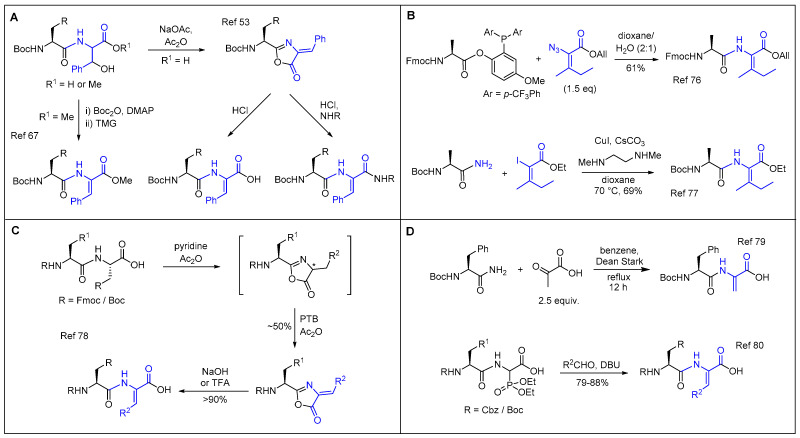
(**A**) Examples of synthesis *via* β-elimination. (**B**) Examples of synthesis via Staudinger ligation and Cu(II)-mediated coupling. (**C**) Examples of synthesis via azlactone oxidation. (**D**) Examples of synthesis via condensation with pyruvic acid and Horner–Wadsworth–Emmons (HWE) olefination.

Many groups, including our own [74], have accessed dehydrodipeptides via a β-elimination strategy, with the initial step involving the conversion of a β-hydroxy group to a better leaving group, which is then followed by a base-induced elimination, often *via* a alkylidene-substituted azlactone intermediate if the *C*-terminus is present as the free acid (Scheme 1A) [60]. In complex syntheses of large peptides, a dehydroamino acid residue is often introduced to solid-phase synthesis as part of a pre-formed dehydrodipeptide, where the dehydroamino acid residue is present at the *C*-terminus. Therefore, the solution phase strategies employed to synthesize these smaller units can be exploited to make the short peptides present in supramolecular dehydropeptide hydrogelators. With this in mind, the Staudinger ligation routes [83] and Cu(II)-mediated coupling routes [84] developed by Inoue, in a route to yaku’amide, may well prove useful in the synthesis of molecules able to form supramolecular structures (Scheme 1B). These methods have the advantage that the *E*/*Z* stereochemistry is pre-installed, albeit slightly eroded during the coupling reaction.

Wołczański and Lisowski recently reported a useful method where a dipeptide azlactone intermediate is oxidized by pyridinium tribromide, which effectively allows canonical amino acids to be converted to their dehydro- versions (Scheme 1C) [85]. More classical methods involve condensation reactions, such as the reaction of Boc-l-Phe-NH_2_ with pyruvic acid [86] or Horner–Wadsworth–Emmons reactions of α-phosphonates with aldehydes (Scheme 1D) [87].

## 4. Conclusions

In this review, we have demonstrated how dehydropeptide supramolecular hydrogels and nanostructures have great potential for many biological applications, particularly as targeted and sustained drug delivery vehicles, as wound treatment materials, and as platforms for 3D cell culture with tissue engineering potential. The presence of a non-proteinogenic dehydroamino acid residue greatly increases resistance to endogenous proteases. Unlike other commonly employed peptidomimetic residues, such as D-amino acids and β-amino acids, dehydroamino acids also decrease the molecular flexibility, which often has a significant effect on the overall secondary structure.

In a general sense, we have seen how dehydropeptides often possess their own intrinsic biological properties, such as anti-bacterial, anti-cancer, anti-inflammatory, and β-breaker activities. Therefore, future work in this area may look to adapt the structures of these biologically active peptides towards hybrid materials with hydrogelation ability. These chimeric materials would then be suitable for “self-delivery” of their own biological properties, perhaps in addition to the encapsulation and then targeted release of other drug molecules. This may be further combined with conjugated drug pharmacophores and/or specific receptor ligands in multi-modal therapies.

Dehydroamino acids, particularly those containing dehydroalanine, can be reactive at C-β to various thiol nucleophiles, through conjugate addition (thia-Michael) reactions. This can be both an advantage and a disadvantage, depending on the system under study. The putative Michael addition will covalently attach the peptide to other biological molecules containing amines and thiols. If this Michael addition is unwanted, then increasing the steric bulk around the double bond reduces the reactivity. In contrast, sometimes, an in vivo conjugation can be specifically targeted. Therefore, short dehydropeptide mimetics of known enzyme inhibitors, capable of hydrogelation, should be investigated as targeted alkylating inhibitors of specific enzymes.

The future direction of dehydropeptide hydrogels should also look to incorporate the aforementioned properties into stimuli-responsive dehydropeptides to form so-called “smart materials”. There are many known methods for either forming or disassembling a hydrogel “on demand”, such as through the application of light, magnetic field, enzymatic action, or pH change. Such strategies can allow the release of drug cargo with spatial and temporal control, can “uncage” an intrinsic biological function, or can allow the facile removal of hydrogel-based wound dressings [88,89,90]. As well as the a

**Figure 8:**
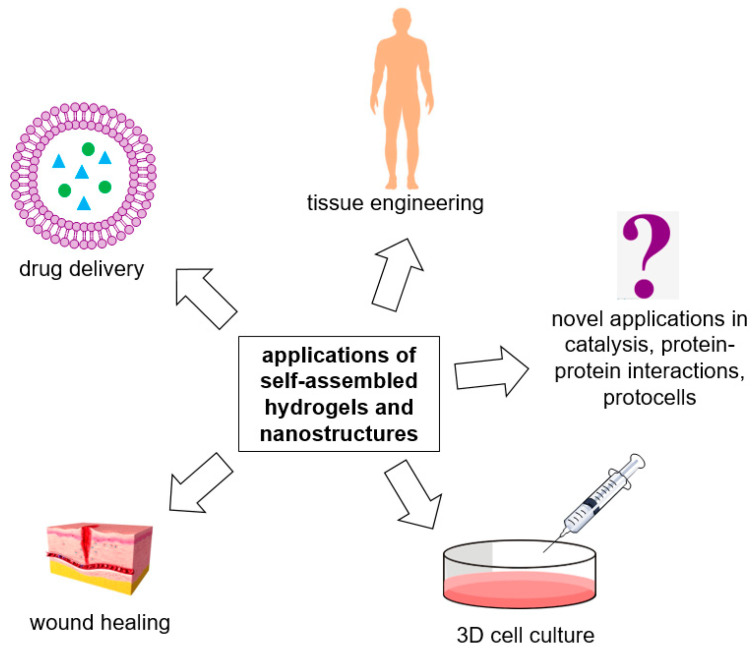
Summary of current and possible future direction of self-assembled hydrogels and nanostructures.

Considering the low toxicity, ease of synthesis, and favorable mechanical and biological properties, we expect to see more of these types of nanostructures developed in the clinic. The required properties of a particular hydrogel/nanostructure may vary, in terms of proteolytic resistance and structural flexibility, depending on the intended biological application. This may dictate if a dehydropeptide is the right choice of building block for a particular purpose. Having discussed their advantages and limitations, we consider the dehydropeptides discussed in this review as complementary to other specific peptide/amino acid motifs, as part of the structural toolbox within the wider area of supramolecular nanostructures. The area of low molecular weight peptide hydrogels has enjoyed incredible growth over the last two decades, from a curiosity of materials science to successful biomedical tools. Research in the areas of drug delivery, tissue engineering, wound dressings, 3D printing, and cell culture will no doubt continue for the foreseeable future, perhaps alongside new applications still to be discovered (Figure 8). New applications may well expand into the food, cosmetics, and arts industries. Possible applications as synthetic nanofactories or even as model biological cells can also be envisaged. The realization of these possibilities is clearly going to require the joint effort of materials chemists, nanotechnologists, biologists, and many others in this interdisciplinary field.

## Abbreviations

Ac: acetyl; AFM, atomic force microscopy; εAhx, 6-aminohexanoic aicd; All, allyl; AMF, alternating magnetic field; Boc, *tert*-butyloxycarbonyl; BSA, bovine serum albumin; Cbz, carboxybenzyl or benzyloxycarbonyl; CGC, critical gelation concentration; COX, cyclooxygenase; DBU, 1,8-diazabicyclo[5.4.0]undec-7-ene; DLS, dynamic light scattering; DMAP, 4-dimethylaminopyridine; ECM, extracellular matrix; FA, folic acid; FAE, *N*-fluoresceinyl-2-aminoethanol; Fmoc, fluorenylmethyloxycarbonyl; Fo, formyl; GdL, glucono-delta-lactone; GSH, glutathione; HFIP, 1,1,1,3,3,3-hexafluoroisopropanol; HWE, Horner–Wadsworth–Emmons; LA, lactobionic acid; LOX, lipoxygenase; Me, methyl; MTT, 3-(4,5-dimethylthiazol-2-yl)-2,5-diphenyl tetrazolium bromide; PTB, pyridinium tribromide; TPGS, D-α-tocopherol polyethylene glycol succinate; SEM, scanning electron microscopy; SPION, superparamagnetic iron oxide nanoparticle; TEM, tunneling electron microscopy; TFA, trifluoroacetic acid; TMG, 1,1,3,3-tetramethylguanidine.
